# Flowability, Tear Strength, and Hydrophilicity of Current Elastomers for Dental Impressions

**DOI:** 10.3390/ma14112994

**Published:** 2021-06-01

**Authors:** Fabian Huettig, Andrea Klink, Alexander Kohler, Moritz Mutschler, Frank Rupp

**Affiliations:** 1 Department of Prosthodontics at the University Clinic for Dentistry, Oral Medicine, and Maxillofacial Surgery, University Hospital Tübingen, Osianderstr. 2-8, 72076 Tübingen, Germany; andrea.klink@med.uni-tuebingen.de (A.K.); moritz.mutschler@frs-nt.de (M.M.); 2 Section Medical Materials Science and Technology, Department of Biomedical Engeneering, University Hospital Tübingen, Osianderstr. 2-8, 72076 Tübingen, Germany; kohleralexander@yahoo.de (A.K.); frank.rupp@med.uni-tuebingen.de (F.R.)

**Keywords:** dental prosthesis, dental materials, dentistry, silicone, dental cast, precision, denture

## Abstract

This study investigates 2 polyethers (PE), 2 polyvinylsiloxanethers (VXSE), and 10 polyvinylsiloxanes (PVS), seven of which had a corresponding light-body consistency and seven of which had a corresponding heavy-body consistency. Each light-body elastomer underwent a flowability test using the shark fin method 20, 50, and 80 s after mixing. The tear strength test DIN 53504 was used after setting the time (T0). Next, 24 h later (T1), hydrophilicity testing was used with static contact angles in water drops during polymerization (20, 50, and 80 s, as well as after 10 min). The heavy-body elastomers underwent shark fin testing with a corresponding light-body material at 50 and 80 s after mixing. The results of light-body testing were combined in a score to describe their performance. The highest differences were detected within flowability in shark fin heights between PE and a PVS (means of 15.89 and 6.85 mm) within the maximum tear strengths at T0 between a PVS and PE (3.72 and 0.75 MPa), as well as within hydrophilicity during setting between VXSE and a PVS (15.09° and 75.5°). The results indicate that VSXE and novel PVS materials can significantly compensate shortcomings in PE towards tear strength and hydrophilicity, but not flowability.

## 1. Introduction

Apart from progress in digitalization and optical acquisition of teeth and jaws, conventional impressions still plays a major role in daily dental practice worldwide. Therefore, improved elastomers are still apart of the dental industry’s R&D focus, as they facilitate precise working casts for restorations and all kinds of dental prostheses, including implants. This is evident through the launch of improved or even novel materials by major companies. Current elastomeric impression materials encompass two prevalent material classes: polyvinylsiloxanes and polyethers [1,2], each of which are supplemented with the “novel” hybrid class polyvinylsiloxanether [3].

Unfortunately, there is insufficient data regarding the current state of elastomers when taking dental impressions. Much of the literature is outdated and cites material characteristics from the late 1990s and early 2000s [4,5]. For elastomers, a large number of properties determine a dentist’s basic decision to use a specific material, which allows its appropriateness in individual clinical cases and broad applications [6].

This addresses a bunch of variables:

- price, storability, and shelf life
- patient comfort (e.g., taste, demolding force)
- available viscosities and their flowability during impressions
- compatibility with astringents or disinfectants
- dimensional stability (over time of transport to the dental lab, reset after compression)
- tolerance towards moisture during impression taking and when pouring the cast
- tear strength to avoid ruptures when demolding from the jaw and/or the gypsum cast

**Figure 1:**
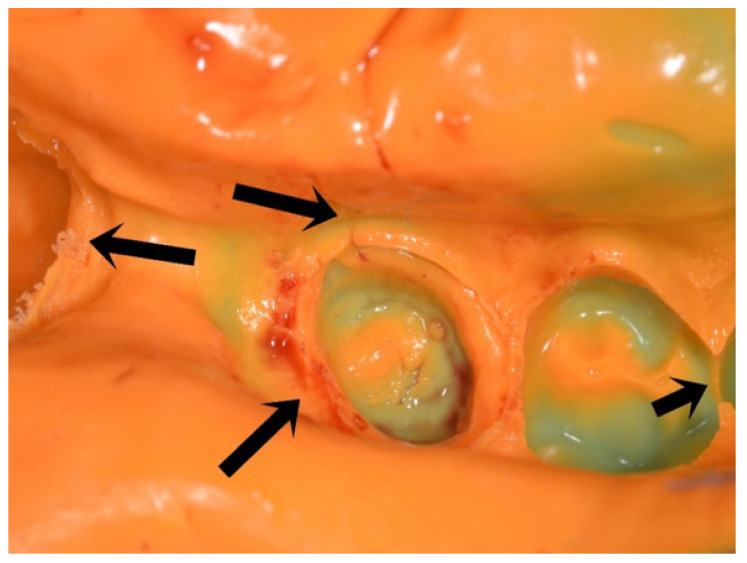
Premolar region of a double mixing impression for a fixed dental prosthesis made from a light-body (orange) and heavy-body (green) polyvinylsiloxane elastomer. The left and right arrow mark ruptures that suffered a loss of material. The top arrow shows a throw of material forming a cleft that intersects the finishing line of the preparation due to an incomplete merging of the material. The lower arrow points at surface changes due to moisture/ blood interaction.

Normally, elastomers shall convey details and dimension without deviations or loss of information from the oral cavity to a working cast. After an impression was taken, the dentist has to visually check the quality of the negative. Dimensional errors are undetectable, but failures or shortcomings appear in reproductions, such as blisters, ruptures, displacement, or incomplete merging (throw) of material (see Figure 1). 

In consequence, the most relevant properties for detailed reproduction are flowability, tear strength, and hydrophilicity. Thus, a material-specific study should evaluate single properties and investigate the most relevant properties from the 14 current elastomers available on the global market (Table 1):

(1) Flowability with regard to the competence to reach and copy complex geometries such as subgingival finishing lines or microtopographical surface features [7].
(2) Tear strength when demolding the impression from the jaw after setting time, as well as 24 h later when the impression demolds from the gypsum cast.
(3) Hydrophilicity with regard to the capacity to flow on dry and moist surfaces during impression taking (initial contact after mixing) [7,8], as well as when the impression is fully polymerized (about 8 min after setting) to be poured in the dental lab.

**Figure 2:**
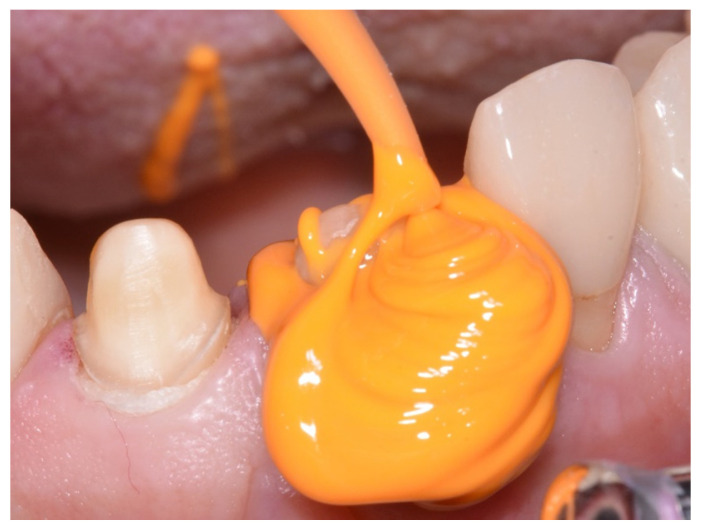
Application of a light-body elastomer to a prepared tooth in the upper jaw. Utilizing the double-mixing technique, the light body material is applied from a cartridge through a mixing canula with a tip directly placed on the hard and soft tissues.

Based on this, we aimed to investigate flowability, hydrophilicity, and tear strength of selected marketed elastomeric impression materials, as well as to evaluate their overall performance with a metric score. Light-body materials were of major interest because they have first contact with the hard and soft tissues and are able to convey their surfaces and details after setting (see Figure 2). 

## 2. Materials and Methods

### 2.1. Elastomers under Observation

The clinical setting of a double mixing impression technique was used to investigate heavy-body tray materials and light-body wash materials (Table 1). Since light-body materials are in instant contact with hard and soft tissues (followed by the medium or heavy-body tray material) their behavior towards flowability, hydrophilicity, and tear strength was preferably tested. 

### 2.2. Testing of the Flowability

**Figure 3:**
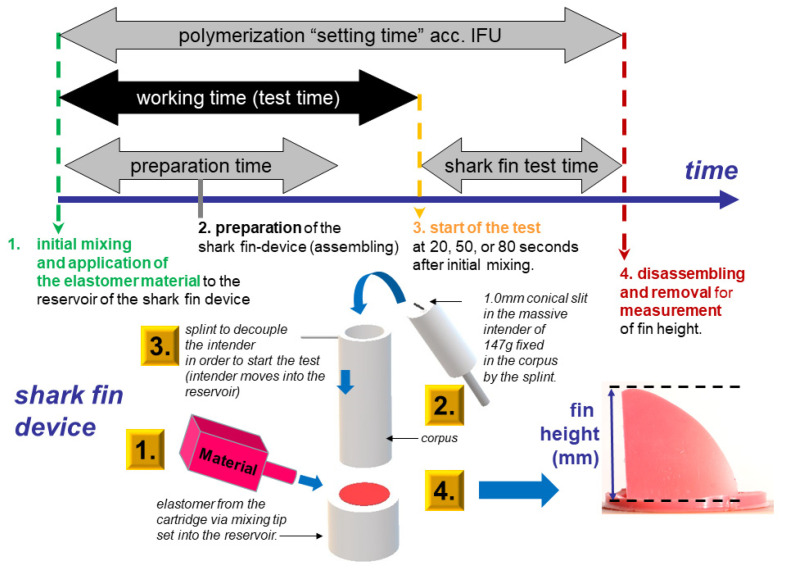
Schematic figure of shark fin testing set up and execution adapted from Huettig et al. [9].

The shark fin testing of the elastomers was facilitated as described by Huettig et al. [9]. The testing device was set up for each elastomer and point in time (Figure 3).

Each material was tested alone (single testing) at 20, 50, and 80 s after their initial mixing. Corresponding heavy and light-body materials were further tested in viscosity combinations (ratio 1:1) at 50 and 80 s after initial mixing (mixed testing). Fin heights were measured blind with a caliper three times. The mean was used as a final height value (in mm) per fin. Each test setting was performed eight times. In total, 21 study groups (14 single, 7 mixed) were established with N = 8 specimen per time set (3 single, 2 mixed).

### 2.3. Testing of the Tear Strength

The tear strength of light-body materials was performed according to DIN 53504:2017 with the “S2 dumbbell specimen”, which was recommended by the technical statute [10]. The specimen had a thickness of 2 ± 0.2 mm and a total length of 75 mm with a bar that was 25 mm long. The heads were 12.5 mm wide and the bar was 4 ± 0.5 mm wide.

**Figure 4:**
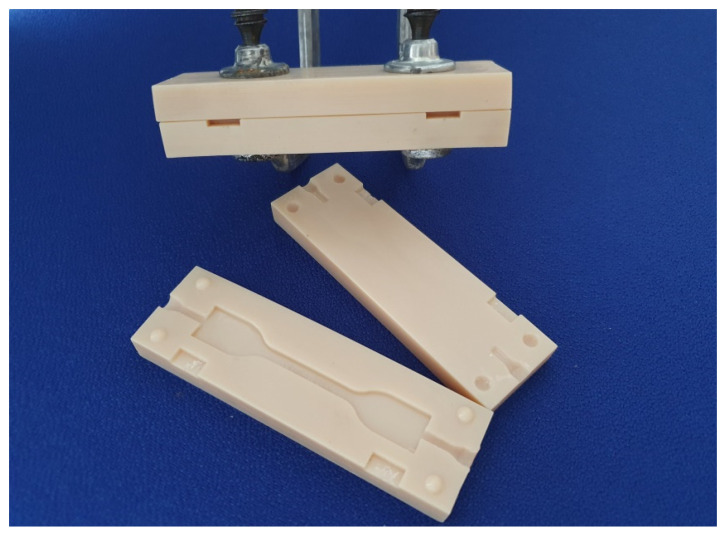
3D-printed injection mold for the S2 dumbbell specimens (For S2 dumbbell Injection mold please refer to Supplementary Materials). The light-body materials were injected directly after mixing in a closed position (fixed with two screw compressor clamps for tight fit). Moreover, 30 s prior to the end of setting time, the tool was opened to remove the dumbbell specimen for the tear strength testing procedure. For specifications and object availability, see Section 2.3.

The dumbbell specimens were fabricated via an injection mold that was 3D-designed and additively manufactured using the PolyJet technique (Objet30 OrthoDesk printing VeroGlaze MED620, both Stratasys Corp., Rechovot, Israel) with 28 µm layer thickness and glossy surfaces (see Figure 4). Therefore, the printing parameters were set to an isometric uniform scale and supports were selected as “standard” in the printing software (Objet Studio, Stratasys Corp.) The injection mold was provided as an STL file when downloaded as a digital object: 10.5281/zenodo.4611959.

**Figure 5:**
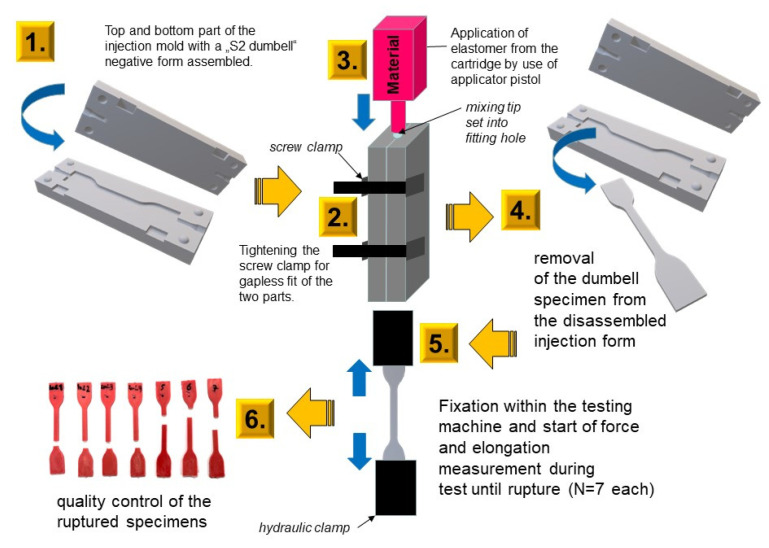
Specimen preparation and testing of the tear strength.

Each study group consisted of N = 7 specimens per time set (T0 = at the end of the setting time (Table 1), T24 = 24 ± 2 h later). The testing device (Z020, Zwick-Roell GmbH, Ulm, Germany) was equipped with a force absorber (500 N), a strain meter with (VideoXtens), and a strain measurement. Wedge clamping elements were hydraulic with a distance of 50 mm. Further, they had a 0.1 MPa initial tensile load when the initial testing distance was 20 mm. The speed level of testing was 500 mm per minute and all tests were performed at 23 °C in 50% relative humidity. The tear strength σ_max_ (MPa), percentage elongation at break eR (%), and tensile strength at 50% elongation σ50 (MPa) were calculated as given in the norm (Figure 5).

### 2.4. Testing of the Hydrophilicity

**Figure 6:**
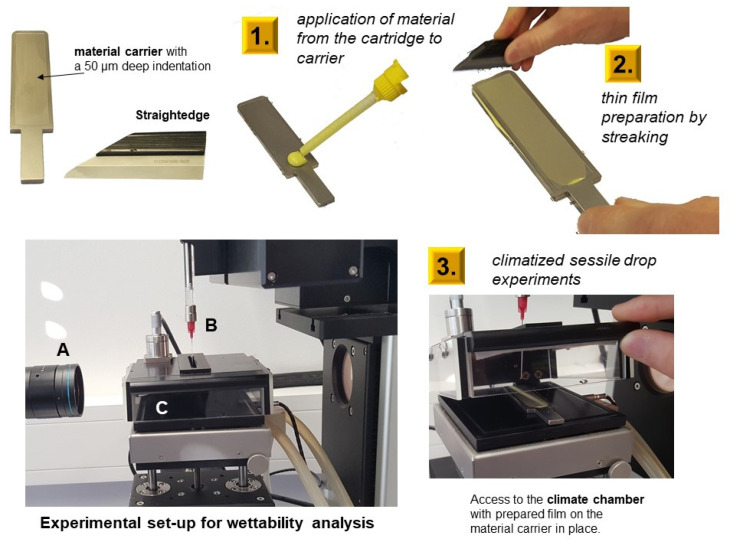
Specimen preparation and testing of hydrophilicity under 80% relative humidity. The wettability analysis set up contained a high speed video camera (**A**), microdrop dosage system (**B**), and climate chamber for specimen presentation (**C**).

Wetting properties of light-body materials were analyzed on unset (20, 50, and 80 s after mixing) and set (600 s after mixing) material surfaces via water contact angles. The storage and measuring temperature was 22 °C. Following Figure 6, the testing was performed in 80% relative humidity using a climate chamber during contact angle measurements (TPC160, DataPhysics Instruments GmbH, Filderstadt, Germany), which was performed with a drop shape analysis system (OCA200 with SCA software, DataPhysics Instruments GmbH, Filderstadt, Germany). The materials were mixed using intraoral tips according to the manufacturers’ instructions. A 50 µm-thick unset of material films were prepared in a corresponding metal mold, which applied a stainless-steel straightedge. After pre-defined times, 2 µL water drops (deionised water) were set onto the surface (Figure 6).

Drop setting and drop shape development were video-controlled and had a framerate of 25/s for 30 s. Each situation (e.g., material, time after mixing) was repeated fivefold (N = 5). Contact angles were defined as angles in a two-dimensional drop-shape evaluation, wherein three-phase points (solid–liquid–gas) were established between the tangent at the respective drop and the horizontal baseline at the material surface [11]. These were analyzed with drop shapes of 1 s, 2 s, and 3 s after the first water drop surface contact (t = 0 when first sharp pic was dropped on the surface). In further calculation, the average of these three measurements was used as a surrogate contact angle to describe hydrophilicity.

### 2.5. Statistical Analysis and Sum Score

The distribution of the obtained variables were initially checked against a normal distribution using Shapiro–Wilk Test (α = 0.05). In case of normality, the means were compared using a one-way analysis of variance (ANOVA) and Tukey’s post-hoc test. Otherwise, a Wilcoxon signed-rank test was applied for multiple comparisons. All comparisons were run with an α of 0.05 under that the null hypothesis assumption that no difference was present between the groups. A connecting letter report was written to group non-statistically significant distributions from each other. Differences were considered statistically significant when the p value was less than 0.05. The following groups were defined for comparison: light-body materials, heavy-body materials, mixed materials, and points in time after mixing or setting.

For a performance cluster, the ranks “R” of each light-body material was summarized as a final score independently from their material group. Therefore, each measurement distribution was not statistically significantly different from other distributions in this measurement group (connecting letter report), which were assigned with a rank, R (connecting letter A = 1, B = 2, etc.). The ranks gained in each experiment were summarized and divided by the number of experiments. This offered a score with a metric measurement (Equation (1)).

(1) $$score=\frac{(\begin{array}{c} \frac{R\;\left[\mathrm{flowability}\right]\left(\mathrm{at}\;20\;s\;+\;\mathrm{at}\;50\;s\;+\;\mathrm{at}\;80\;s\right)}{3}\\ +\frac{R\;\left[\mathrm{contact}\;\mathrm{angle}\right]\left(\mathrm{at}\;20\;s+\;\mathrm{at}\;50\;s\;+\;\mathrm{at}\;80\;s\;+\mathrm{at}\;600\;s\right)}{4} \end{array})+\left(\begin{array}{c} \frac{R\;\left[\sigma_{\max}\;T0\right]\left(\mathrm{at}\;20\;s\;+\;\mathrm{at}\;50\;s\;+\;\mathrm{at}\;80\;s\right)}{3}\\ +\frac{R\;\left[\sigma_{\max}\;T24\right]\left(\mathrm{at}\;20\;s\;+\;\mathrm{at}\;50\;s\;+\;\mathrm{at}\;80\;s\right)}{3} \end{array}\right)÷2}{3}$$

Equation (1) demonstrates a formula to calculate the performance score out of the ranks “R” from the single experiments (e.g., flowability at 20 s). A rank is derived from the connecting letter report of the post-hoc statistical testing of distributions.

## 3. Results

### 3.1. Flowability

All materials were suitable to form fins within the test set up at all times after initial mixing. The mean fin heights and ranks (R) were derived from statistically different groups (Table 2, Table 3 and Table 4).

### 3.2. Tear Strength

The polymerized specimen could be successfully tested in pre-set time frames. The tear strength values at T0 and T24 are given in Table 5. The rupture was located near the radius of the specimens tested at T24 in AqL (n = 4); FiS (n = 1); FtC (n = 4); IL4 (n = 3); IdL (n = 6); ImL (n = 4); and PaL (n = 2). This is especially true when compared to T0, which was only observed in FtC (n = 1) and ImL (n = 3).

**Figure 7:**
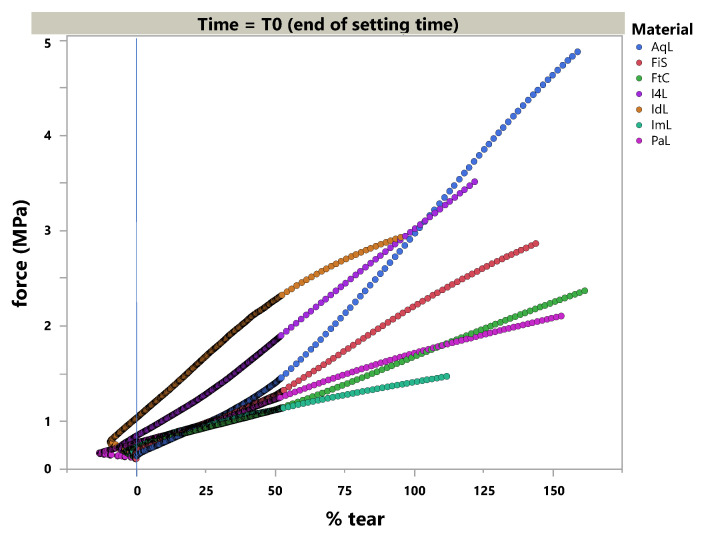
Stress–strain diagram of the light-body materials at the end of setting time (T0.)

**Figure 8:**
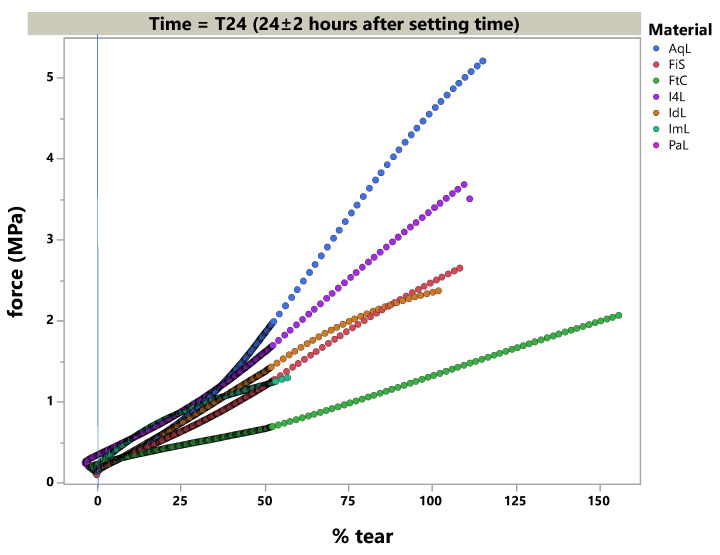
Stress–strain diagram of the light-body materials 24 h after impression taking (T24).

Therefore, the load-elongation curves differed, as shown in Figure 7 and Figure 8.

### 3.3. Hydrophilicity

**Figure 9:**
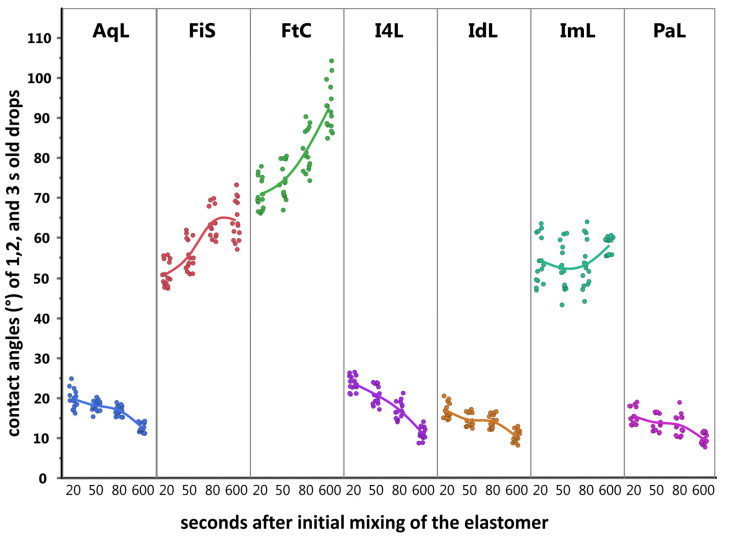
Wettability behavior over time. Three measured contact angles (y-axis) met at the polymerizing light-body materials after initial mixing (x-axis). Each dot gave one angle measurement at the drop. Scattering of the dots offered insight into spreading behavior of the drop from 1 to 3 s (15 measurements from 5 specimens per group).

Table 6 shows the contact angles received from the light-body materials based on the sessile drop technique. The time dependent changes are illustrated in Figure 9.

### 3.4. Performance Analysis of Light-Body Materials

**Figure 10:**
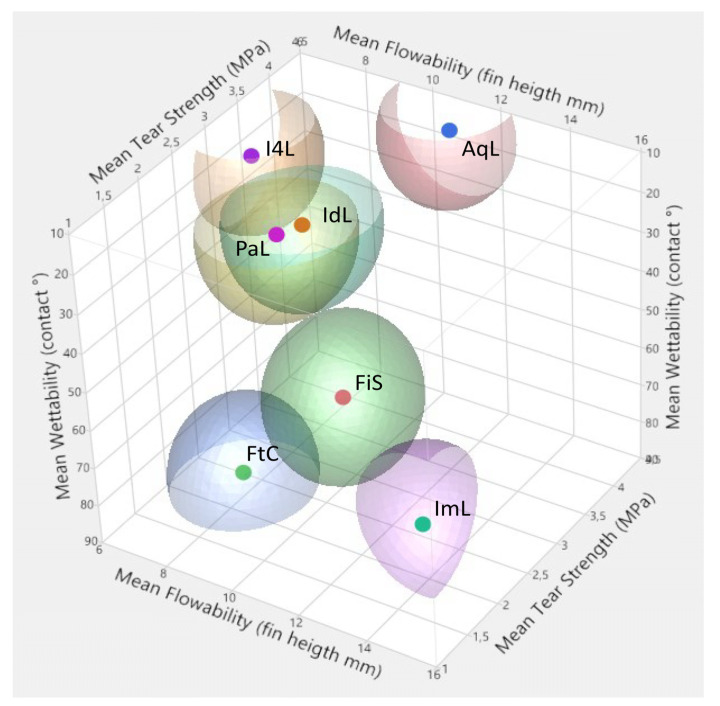
Data illustration that indicates how materials orient around the three tested properties: tear strength, flowability, and wettability.

The mean ranks derived from the three experiments, as well as the final score, are shown in Table 7. Figure 10 visually interrelates the measured mean values of flowability (fin height), hydrophilicity (contact angle), and tear strength (σ_max_), specifically with regard to light-body materials.

## 4. Discussion

Because there are many types of impression materials on the market, it is difficult to survey all of the currently used properties [4]. Since 2010, the research terms “impression material” AND “dental” have dropped from about 3 out of 1000 scientific manuscripts to about 2 out of 1000 manuscripts, ranging at 2.37 per 1000 a year (see Appendix A).

As mentioned in the introduction, conventional impressions are still necessary in dental practice, especially vis-à-vis full arch rehabilitations including prosthetic treatment of multiple implants [12]. This process includes detailed reproduction of subgingival areas (not detectable by scanning technologies). Therefore, flowability, wettability, and tear strength are of major academic and practical interest [4].

### 4.1. Flowability

Flowability is strongly connected to an elastomer’s viscosity. Light-body materials have high viscosity, but their ability to flow under pressure when clinically applied in a tray can differ. Several approaches can be used to test rheological properties such as the application of a rheometer to reveal viscosity (Pa s), elastic modulus (kPa), and tan delta to reveal polymerization over time [7]. Flowability also addresses thixotropic behavior [13].

Shark fin testing offers a device that is easier to use and comprehend the results. However, the test is limited to one time point per test. One may assume that this was why it has been frequently applied to compare flowability under a standardized weight [7,9,13,14]. However, the comparison of reported fin heights has been proven to be hindered by the test environment, such as slit width, testing time after initial mixing, room and material temperature, the mode of application (single vs. layered), and dimensions of the applied device itself [15]. Nevertheless, it allows for a comparative view of the tests, which can be conveyed to other tests. As detected by Rupp et al., the clinical time of application is around 80 s after initial mixing [16].

PE forms have been found to possess the highest fins at 80 s. PVXE and PVS materials possess the second highest fin at 80 s. These findings align with the findings presented in this study [7,9,14,17]. This implies that PE may outperform subgingival structures and have an increased risk of cleft formations (as shown in Figure 1) due to its higher tendency to merge while flowing under pressure. Here, PE resulted in constantly high fins (about 15 mm) during working times (20, 50, and 80s). On the other hand, VSXE showed a slight drop (−13.9%) from 20 to 80 s and PVS dropped between −21.7% and −67.6% from the initial value. PVS materials also showed a high spread of flowability 80 s after initial mixing, resulting in a 9 mm to 3.5 mm fin height. Consequently, in case of routine shortcomings (e.g., clefts, unrecorded areas) the clinician should reflect the potential influence of flowability to the applied PVS. The PVXE materials may offer a clinical compromise in flowability, as described by Enkling et al. [18].

Finally, one should take into account that a ”higher” shark fin “is not necessarily the prerequisite for high dimensional accuracy and good surface detail reproduction of the clinical impressions, respectively” [17].

### 4.2. Tear Strength

High tear strength is a favorable property in elastomers as it enables the clinical avoidance of ruptures and scissures for thin flags that could likely be formed with a retracted sulcus or induced by any interdental space due to the absence of a papilla after the tray is removed from the jaw. In a dental lab setting, when the impression is removed from the cast, a rupture in the impression material may align with a loss of areas that cover the finishing line of abutting teeth. This hinders a second cast fabrication (control cast) of the same detail and validity.

To simulate the clinical situation of demolding, grip separation was set to 500 mm per minute (=8.3 mm/s), which should imitate sulcus acceleration in the moment of removal. Lawson et al. showed that a higher crosshead speed (500 mm vs. 1 mm per minute) tendentially resulted in higher tear strength values directly after polymerization and 24 h later. Moreover, their test setting revealed two to three fold higher values (ranging from about 2–8 MPa) for PE, PVXE, and PVS due to different specimens [19]. Testing according to the statute for elastomers revealed small standard deviations (comparable to other studies), but it also failed specimens due to their impermissible rupture mode. This could be attributed to trapped air or merging failures within the injection mold. This observation may offer further research opportunities for the flowing and merging behaviors of elastomers. Pandey et al. used 50 mm/min and found comparable behavior in the order of tear strength values, i.e., PE < VSXE < PVS. However, they did not report the testing time [20]. This shows that, over the past four decades, PVS tear strength improved over time compared to PE [21], and outperformed by one specific material in this study (AqL).

In summary, the tear strength of PVS and VSXE materials was found to be superior to PE materials.

### 4.3. Wettability

Liquids contacting solid materials and how they spread after contact can be characterized and quantified by contact angles. Considering the thermodynamic nature of energetic surface calculations and the so-called Young contact angle, a liquid’s drop shape on solid surfaces can form as a result of different interfacial energies [11]. Besides thermodynamic rules, this is useful in non-ideal inhomogeneous systems to measure apparent contact angles even on non-solid, unset material films. This is because they can be used as an indicator of wettability or hydrophilicity (if water is the liquid used). Several studies have shown the appropriateness of experimental approaches that used sessile drop measurements on set and unset polymerizing elastomeric materials [16,22,23]. The concept of using so-called initial but unequilibrated contact angles soon after contact has been proven useful for characterizing the hydrophilic state of an impression material when it first contacts an oral surfaces. In the current study, a climate chamber was used to simulate oral relative humidity, which has been shown in an earlier study to possibly influence wetting properties of impression materials [24]. In this study, two PVS materials (FiS and FtC) showed a loss of hydrophilic properties during working time and polymerization, whereas all other materials showed constant or even decreasing contact angles. Three PVS materials and the VSXE material outperformed the polyether (ImL) with significantly lower contact angles, respectively. In this group of four materials, the contact angles ranged between 16.9° (I4L) and 13.2° (PaL) 80 s after initial mixing. This, therefore, characterized the very hydrophilic materials. At this time point, a much lower hydrophilicity was ascribed to ImL, FiS, and especially to FtC, which had the highest mean contact angle of 81.6°. Similarly to the unset situation, set impression materials can be subdivided into a very hydrophilic group, a moderate hydrophilic group, and a low hydrophilic group. The latter group is formed by FtC, which has a 92.5° mean contact angle in the set state and is the only material that exceeds the 90-degree boundary that separates hydrophilic from hydrophobic surface properties. Moreover, the moderate yet constant hydrophilic characteristic is a typical wetting behavior of polyethers due to their hydrophilic chemical structure. Earlier studies often showed a reduction of their initial hydrophilicity in PVS. This was because the material was in a hydrophobic state at the end of the working time, indicating that the active surface was more limited due to its limited molecular mobility in an almost polymerized state [16]. The current study shows that the current generation of PVS materials remains hydrophilic, even though some materials have become less hydrophilic.

In summary, the general “hydrophobic character” of PVS was found to only be valid for a few materials [4].

### 4.4. Limitations

This study investigated three major properties of impression materials. Our findings are of clinical relevance and their impact is visually detectable by clinicians after taking an impression. However, this study excluded the following considerations: detailed reproduction under dry and wet conditions; dimensional stability (i.e., shrinkage and swelling) in dry and wet conditions, as well as thermal expansion behavior; compression and reset; stickiness and interaction with astringents or patient-relevant variables, such as comfort or taste. Furthermore, some clinical circumstances that may interact with the evaluated properties was lacking due to standardization. More specifically, we were unable to verify the impact of temperature on flowability and wettability, or the effect of disinfection and disinfectants after polymerization on wettability and tear strength [25].

## 5. Conclusions

Current elastomers show favorable yet diverging properties (i.e., flowability, wettability, and tear strength). Current PVS and VSXE provide wetting behavior that is superior to PE. Here, tear strength σ_max_ ranged between 2 and 3 MPa for PVS and VSXE after setting times. It also underwent less than 1 MPa for PE. This could have caused a higher risk of ruptures in PE when recording subgingival areas because it had a higher flowability than VSXE and PVS. The clinical PVS was relevant to these properties and thus should be critically reflected by observations in dental practice, as well as investigated in academia. Based on these results, we hypothesize that impression materials that show an overall “good” score in all properties might be clinically superior to materials with a “very good” score for only one characteristic.

In summary, our results indicated that VSXE and novel PVS materials were capable of compensating for the shortcomings of PE, specifically towards tear strength and hydrophilicity, but not flowability.

## Figures and Tables

**Table 1:** Overview of elastomer properties and the performed tests. Material groups: PE = polyether, PVS = polyvinylsiloxane, PVXE = polyvinylsilaxonether. Viscosity: L = light-body, H = heavy-body. Tests: F = flowability using shark-fin; T = tear strength applying DIN 53504; H = hydrophilicity using contact angle of 1 and 3 s old drops. Corresponding materials for wash impression technique are grouped in one row (e.g., AqH-AqL).

Material Group	Viscosity	Abbreviation Study Group	Materials Brand Name	Manufacturer	LOT#	Exp. Date Year-Month	Processing Time (s)	Setting Time (s)	Tests
PVS	H	AqH	Aquasil Ultra+ Heavy	Dentsply	180103	January 2021	135	300	F
PVS	L	AqL	Aquasil Ultra+ XLV		171017	October 2020	135	300	F,T,H
PVS	H	FtH	Flexitime Heavy Tray	Heraeus Kulzer	K010157	August 2020	150	150	F
PVS	L	FtC	Flexitime Correct Flow		R010045	September 2019	150	150	F,T,H
PVS	H	PaH	Panasil tray Soft Heavy	Kettenbach	170591075	September 2019	120	240	F
PVS	L	PaL	Panasil Initial contact X-Light		170701	May 2020	90	150	F,T,H
PVS	H	SyC	Symmetric Comfort	Kaniedenta	785984	April 2021	105	270	F
PVS	L	FiS	Fitnis SH light		61802062	March 2021	90	210	F,T,H
PVS	H	I4H	Imprint 4 Penta Heavy	3M ESPE	3699084	December 2019	120	120	F
PVS	L	I4L	Imprint 4 Light		3944024	February 2020	60	120	F,T
PVS	L	I4L	Imprint 4 Light		3714170	December 2019	60	120	H
PE	H	ImP	Impregum Penta H Duosoft	3M ESPE	3830139	July 2020	150	360	F
PE	L	ImL	Impregum Garant L DuoSoft		3859100, 3737581	October 2019, August 2019	120	330	F,T,H
VSXE	H	IdH	Identium Heavy	Kettenbach	170541034	July 2019	120	270	F
VSXE	L	IdL	Identium Light		170191, 180211	February 2019, December 2019	120	150	F,T,H

**Table 2:** Mean and SD of fin heights were derived after testing light-body materials and their ranks (R). Tests had a statistical difference within each time group.

Time after Inital Mixing (s)	Light-Body Materials																				
	AqL			FiS			FtC			I4L			IdL			ImL			PaL		
	Mean	SD	R	Mean	SD	R	Mean	SD	R	Mean	SD	R	Mean	SD	R	Mean	SD	R	Mean	SD	R
20	12.12	0.62	2	15.01	0.34	1	9.45	0.45	4	10.82	0.31	3	10.99	0.69	3	15.59	0.62	1	12.81	0.53	2
50	10.84	0.62	2	10.35	0.39	2	8.31	0.46	3	6.23	0.27	5	10.18	0.51	2	15.92	0.81	1	9.92	0.58	3
80	9.04	0.25	2	6.94	0.74	3	7.40	0.31	3	3.51	0.20	5	9.46	0.28	2	15.56	0.27	1	7.29	0.39	3
average	10.67	1.39	2	10.77	3.42	2	8.39	0.95	3.3	6.85	3.09	4.3	10.21	0.81	2.3	15.69	0.60	1	10.01	2.35	2.3

**Table 3:** Mean and SD of fin heights were derived after testing heavy-body materials and their ranks (R). Tests had a statistical difference within each time group.

Time after Inital Mixing (s)	Heavy-Body Materials																				
	AqH			FtH			I4H			IdH			ImP			PaH			SyC		
	Mean	SD	R	Mean	SD	R	Mean	SD	R	Mean	SD	R	Mean	SD	R	Mean	SD	R	Mean	SD	R
50	2.35	0.05	2	0.79	0.08	6	1.80	0.08	3	1.72	0.07	3	5.11	0.11	1	1.07	0.08	5	1.57	0.11	4
80	1.52	0.15	2	0.48	0.07	6	1.20	0.08	3	0.96	0.09	4	4.98	0.08	1	0.67	0.03	5	0.88	0.04	4
average	1.94	0.44	2	0.64	0.18	6	1.50	0.32	3	1.34	0.40	3.5	5.05	0.11	1	0.87	0.21	5	1.23	0.36	4

**Table 4:** Mean and SD of fin heights were derived after testing the mixed corresponding light and heavy-body materials and their ranks (R) within each time group.

Time after Inital Mixing (s)	Corresponding Materials																				
	AqH-AqL			FtH-FtC			I4H-I4L			IdH-IdL			ImP-ImL			PaH-PaL			SyC-Fis		
	Mean	SD	R	Mean	SD	R	Mean	SD	R	Mean	SD	R	Mean	SD	R	Mean	SD	R	Mean	SD	R
50	10.04	0.50	3	8.22	0.98	4	7.71	0.32	5	9.43	1.33	3	16.43	0.46	1	9.96	1.25	3	11.87	1.21	2
80	8.97	0.32	2	8.12	0.59	2	4.55	0.17	4	8.56	0.84	2	15.66	0.63	1	7.02	0.69	3	6.75	0.70	3
average	9.50	0.69	2.5	8.17	0.78	3	6.13	1.65	4.5	8.99	1.16	2.5	16.04	0.66	1	8.49	1.80	3	9.31	2.81	2.5

**Table 5:** Results from the tear strength measurements of the light-body materials at the end of the setting time (T0) and 24 h later (T24). Ranks had a statistical difference within each time group.

Material	T0			T24			T0			T24			T0			T24		
	σ_50_ (MPa)			σ_50_ (MPa)			eR (in %)			eR (in %)			σ_max_ (MPa)			σ_max_ (MPa)		
	Mean	Std Dev	R	Mean	Std Dev	R	Mean	Std Dev	R	Mean	Std Dev	R	Mean	Std Dev	R	Mean	Std Dev	R
AqL	0.75	0.12	2	1.92	0.17	1	197	28.41	1	108.95	8.85	3	3.72	0.39	1	5.05	0.32	1
FiS	0.78	0.05	2	1.20	0.08	4	171	39.23	1	116.99	9.85	2	2.30	0.29	3	2.64	0.12	3
FtC	0.65	0.04	2	0.68	0.04	5	201	25.35	1	183.96	28.91	1	2.07	0.23	3	2.03	0.19	4
I4L	1.35	0.08	1	1.66	0.09	2	118	5.77	2	102.60	8.37	3	3.00	0.18	2	3.49	0.14	2
IdL	1.38	0.25	1	1.44	0.05	3	122	25.69	2	117.13	9.39	2	2.21	0.26	3	2.40	0.07	3
ImL	0.52	0.10	3	1.15	0.09	4	116	36.71	2	85.58	19.80	3	0.75	0.14	5	1.41	0.14	5
PaL	0.81	0.17	2	1.18	0.06	4	179	33.03	1	137.41	14.67	2	1.82	0.27	4	2.21	0.09	4

**Table 6:** Results from the contact angle measurements (in °) was used to determine the wetting behavior. Ranks had a statistical difference within each time group.

Material	Time after Initial Mixing (s)												
	20			50			80			600 + Setting Time			
	Contact Angle (°)			Contact Angle (°)			Contact Angle (°)			Contact Angle (°)			
	Mean	Std Dev	R	Mean	Std Dev	R	Mean	Std Dev	R	Mean	Std Dev	R	Mean R
AqL	19.76	2.40	2	18.01	1.30	2	16.82	1.22	2	12.42	1.17	2	2
FiS	50.97	3.08	3	55.64	3.91	4	64.04	3.92	4	64.33	5.05	4	3.75
FtC	70.75	4.05	4	74.11	4.36	5	81.64	5.17	5	92.53	5.97	5	4.75
I4L	23.62	1.86	2	20.69	2.32	3	16.94	2.15	2	11.10	1.53	2	2.25
IdL	16.80	1.89	1	14.42	1.67	1	14.05	1.62	1	10.30	1.48	1	1
ImL	54.38	5.89	3	52.35	5.62	4	53.32	5.96	3	58.09	2.10	3	3.25
PaL	15.47	2.10	1	13.75	2.03	1	13.18	2.55	1	9.74	1.39	1	1

**Table 7:** Light-body materials rankings from the three experiments, sorted by the final rank (sum score). Green, yellow, and red indicate a score classification of ≤2.5, ≤3.5, and >3.5. * The flowability rank derives from the sum of the mean rank values from light-body materials and the mixed test set up divided by 2.

Elastomer	Flowability *	Hydrophilicity	Tear Strength	Sum Score (Final Rank)
AqL	2.17	2	1.5	5.67 (1)
IdL	2.77	1	2.33	6.10 (2)
PaL	3.43	1	2.83	7.26 (3)
ImL	1	3.25	3.66	7.91 (4)
I4L	3.93	2.25	2	8.18 (5)
FiS	2.83	3.75	2.5	9.08 (6)
FtC	4.1	4.75	2.67	11.52 (7)

## Data Availability

Measurement data are available without restrictions upon request.

## Supplementary Materials

Injection mold of S2 dumbell for download as digital object in STL is available via: https://doi.org/10.5281/zenodo.4611959.

## Appendix A

**Table A1:** Annual results when searching “impression material” And “dental” in Pubmed Library.

Year	“Dental” (N Results)	“Impression Material” AND “Dental” (N Results)	Share Out of 1000 Results (‰)
2010	16,001	53	3.31
2011	18,248	44	2.41
2012	19,949	60	3.01
2013	21,386	62	2.90
2014	23,378	62	2.65
2015	24,964	54	2.16
2016	25,290	47	1.86
2017	25,556	45	1.76
2018	26,075	55	2.11
2019	26,871	61	2.27
2020	30,737	49	1.59
